# Supplementary material for: On fuzzy Henstock-Stieltjes integral on time scales with respect to bounded variation function
Source: PLoS One. 2024 Sep 26;19(9):e0309031. doi: 10.1371/journal.pone.0309031 (PMC11426518; doi:10.1371/journal.pone.0309031)
Supplement: Attachment — Submitted filename: Report on PLOS-ONE-D-24-12527.pdf [file pone.0309031.s001.pdf]

Report on: On Fuzzy Henstock-Stieltjes Integral on Time Scales with respect to Bounded Variation Function

The considered problem is interesting, and the results are proven with the standard method. However, the representation of the paper is not satisfactory. It requires some revision. I have some suggestions and comments:

1. There are many typos and grammatical errors throughout the paper, especially in the introduction section.
2. Use a mathematical environment for "inf," "sup," "max," "min," etc.
3. The authors have included many preliminaries. It would be beneficial if they provided a brief explanation of their relevance to the results.
4. Cite Definition 3.1 and others.
5. There are insufficient smooth connections between the results. It is recommended that the authors provide a few introductory lines before stating and proving each new result.
6. If possible, could the authors include any corollaries and remarks for T=R and T=Z?
7. The results are applicable to general time scales and therefore could be relevant to both continuous and discrete time scales. It would be beneficial to compare these results with existing ones on real and discrete time scales, if possible.
8. The conclusion section is too brief. It should provide a more detailed explanation.
9. The main contribution of this paper is based on the time scales theory; however, the authors did not adequately explain or cite many papers from this area. It is suggested that they include additional relevant references from the literature, such as 10.29020/nybg.ejpam.v11i2.3200; 10.1016/j.jfranklin.2020.11.001; 10.1016/j.apm.2022.12.027; 10.1007/s00521-023-08980-5;10.1016/j.nahs.2020.100986

Based on the above facts, I recommend a major revision.
